# Euglycemic Diabetic Ketoacidosis: Experience with 44 Patients and Comparison to Hyperglycemic Diabetic Ketoacidosis

**DOI:** 10.5811/westjem.60361

**Published:** 2023-09-25

**Authors:** Jordan Sell, Nathan L. Haas, Frederick K. Korley, James A. Cranford, Benjamin S. Bassin

**Affiliations:** * University of Michigan, Department of Emergency Medicine, Ann Arbor, Michigan; † University of Michigan, Department of Emergency Medicine, Division of Critical Care, Ann Arbor, Michigan; ‡ The Max Harry Weil Institute for Critical Care Research and Innovation, Ann Arbor, Michigan

## Abstract

**Introduction:**

Euglycemic diabetic ketoacidosis (DKA) (glucose <250 milligrams per deciliter (mg/dL) has increased in recognition since introduction of sodium-glucose co-transporter 2 (SGLT2) inhibitors but remains challenging to diagnose and manage without the hyperglycemia that is otherwise central to diagnosing DKA, and with increased risk for hypoglycemia with insulin use. Our objective was to compare key resource utilization and safety outcomes between patients with euglycemic and hyperglycemic DKA from the same period.

**Methods:**

This is a retrospective review of adult emergency department patients in DKA at an academic medical center. Patients were included if they were >18 years old, met criteria for DKA on initial laboratories (pH ≤7.30, serum bicarbonate ≤18 millimoles per liter [mmol/L], anion gap ≥10), and were managed via a standardized DKA order set. Patients were divided into euglycemic (<250 milligrams per deciliter [mg/dL]) vs hyperglycemic (≥250 mg/dL) cohorts by presenting glucose. We extracted and analyzed patient demographics, resource utilization, and safety outcomes. Etiologies of euglycemia were obtained by manual chart review. For comparisons between groups we used independent-group *t*-tests for continuous variables and chi-squared tests for binary variables, with alpha 0.05.

**Results:**

We identified 629 patients with DKA: 44 euglycemic and 585 hyperglycemic. Euglycemic patients had milder DKA on presentation (higher pH and bicarbonate, lower anion gap; *P* < 0.05) and lower initial glucose (195 vs 561 mg/dL, *P* < 0.001) and potassium (4.3 vs 5.3 mmol/L, *P* < 0.001). Etiologies of euglycemia were insulin use prior to arrival (57%), poor oral intake with baseline insulin use (29%), and SGLT2 inhibitor use (14%). Mean time on insulin infusion was shorter for those with euglycemic DKA: 13.5 vs 19.4 hours, *P* = 0.003. Mean times to first bicarbonate >18 mmol/L and first long-acting insulin were similar. Incidence of hypoglycemia (<70 mg/dL) while on insulin infusion was significantly higher for those with euglycemic DKA (18.2 vs 4.8%, *P* = 0.02); incidence of hypokalemia (<3.3 mmol/L) was 27.3 vs 19.1% (*P* = 0.23).

**Conclusion:**

Compared to hyperglycemic DKA patients managed in the same protocolized fashion, euglycemic DKA patients were on insulin infusions 5.9 hours less, yet experienced hypoglycemia over three times more frequently. Future work can investigate treatment strategies for euglycemic DKA to minimize adverse events, especially iatrogenic hypoglycemia.

Population Health Research Capsule What do we already know about this issue?

*Euglycemic diabetic ketoacidosis (DKA) is often treated with similar protocols as hyperglycemic DKA.*

What was the research question?

*What are the triggers of euglycemia, and how do treatment and safety outcomes differ between euglycemic and hyperglycemic DKA?*

What was the major finding of the study?

*Euglycemic DKA patients had more than 3 times the rate of hypoglycemia while on insulin infusion: 18.2% vs 4.8% (P = 0.02).*

How does this improve population health?

*Euglycemic DKA can be present in patients who are not taking SGLT2 inhibitors, and these patients are at increased risk of iatrogenic hypoglycemia during treatment.*

## INTRODUCTION

Diabetic ketoacidosis (DKA) is a common and dangerous condition encountered in the emergency department (ED). Severe insulin deficiency triggers increases in counter-regulatory hormones such as cortisol, glucagon, and catecholamines, which results in hyperglycemia and ketoacidosis and requires treatment with exogenous insulin.^1^ Standardized, protocolized treatment with intravenous (IV) fluids, insulin, and electrolyte management have led to vastly improved outcomes; however, this requires prompt identification of the clinical entity and initiation of treatment. In certain cases, the body is unable to mount a hyperglycemic response due to either reduced glucose stores (starvation state, chronic liver disease, heavy alcohol use) or is losing glucose more rapidly than can be produced (sepsis, urinary losses, exogenous insulin).^2^ ^,^ ^3^

This results in a state of euglycemic DKA, which poses a challenge to clinicians, as hyperglycemia, often the first trigger to consider DKA as a potential diagnosis, is not present. It also poses challenges in management with higher attentiveness required to avoid hypoglycemia with use of IV insulin infusion to resolve the ketoacidosis. This condition has gained recognition in recent years given its association with sodium-glucose co-transporter 2 (SGLT-2) inhibitor use, which is only becoming more prevalent as recent American Heart Association guidelines have given a Class 1 recommendation for its use in patients with heart failure.^4^ This will also likely increase the prevalence of euglycemic DKA seen in EDs, which currently comprise only 2.6–3.2% of admissions for DKA.^5^

Most of the literature published to date regarding management of euglycemic DKA is centered on using standardized DKA treatments with IV fluids, insulin infusions, and electrolyte management, with the added caveat that glucose will need to be added to fluids early to prevent hypoglycemia. However, very little has been published on the differences in patient demographics and lab values at presentation, approaches to management, or clinical and safety outcomes between euglycemic and hyperglycemic DKA patients. Moreover, many clinicians have built a strong association of euglycemic DKA with SGLT2 inhibitors such that clinical suspicion may be inappropriately lacking in patients who are not taking one of these medications.

Given the challenges in identifying and treating this clinical entity, we sought to analyze our experience with time-matched cohorts of euglycemic and hyperglycemic DKA patients at our institution, both of which were managed with the same two-bag protocol. We also identified etiologies for euglycemia on presentation as risk factors to heighten suspicion for this condition.

## METHODS

This was a retrospective review of adults in DKA managed in the ED at a single academic medical center in the United States. The Institutional Review Board at the University of Michigan reviewed this study (HUM00224835). This study is presented in accordance with the STROBE (Strengthening the Reporting of Observational studies in epidemiology) statement.

We conducted a retrospective structured chart review of all DKA patients who presented to our adult ED from August 2015–October 2022. A standardized order set for management of DKA was implemented in August 2015, and defining the study period in this fashion promoted the largest sample size possible. Data points of interest were identified and extracted from the electronic health record using an automated query. Patients were included if they were adult (≥18 years old), met diagnostic criteria for DKA based on initial ED laboratory studies (pH ≤ 7.30, serum bicarbonate ≤18 millimoles per liter [mmol/L], anion gap ≥10), and were managed via a standardized DKA two-bag method order set.^1^ ^,^ ^6^ Patients were subdivided into euglycemic DKA (initial glucose ≤250 milligrams per deciliter [mg/dL]) and hyperglycemic DKA (initial glucose >250 mg/dL). Patients were excluded if more than one insulin infusion order set was used (ie, the two-bag method order set plus an additional titratable insulin infusion order set used in other areas of our hospital).

**Figure 1.**
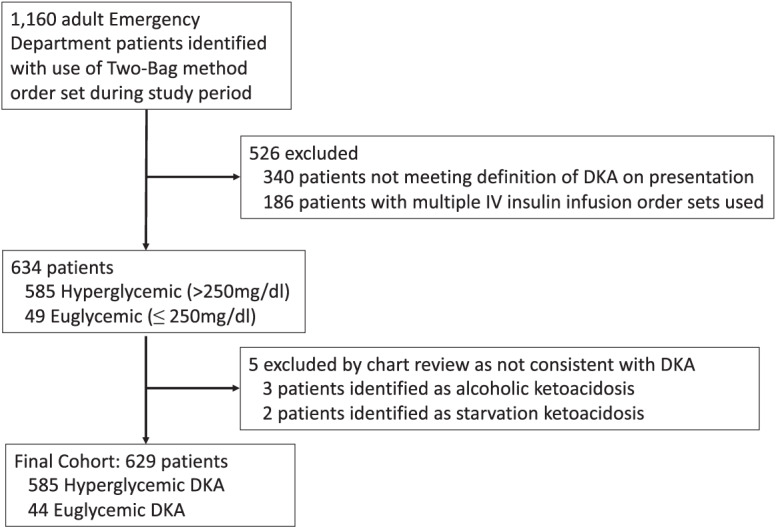
Identification and screening of patients. *IV*, intravenous; *DKA*, diabetic ketoacidosis.

Patients in this study were all managed in an ED-based intensive care unit (ED-ICU).^7^ ^,^ ^8^ Our initial search identified 1,160 adult ED patients managed via the two-bag method. We excluded 340 patients for not meeting DKA laboratory criteria, 186 due to use of multiple insulin infusion order sets, and five after chart review identified alcoholic ketoacidosis or starvation ketoacidosis as their ED diagnosis. Starvation ketoacidosis was differentiated from euglycemic DKA primarily by resolution of ketoacidosis with glucose supplementation and lack of diagnosed diabetes mellitus before or during their presenting illness. This resulted in our final cohort of 629 patients, 44 of whom were euglycemic on presentation and 585 hyperglycemic as outlined in Figure 1.

Age, gender, weight, and initial laboratory values were extracted for patient demographics. We assessed resource utilization data including duration of insulin infusion (defined as the interval from the first insulin infusion start time to the last insulin infusion stop time); time from ED presentation to first pH >7.3; time from ED presentation to first bicarbonate >18 mmol/L; lengths of stay (LOS) in (ED, ED-ICU, hospital, ICU); and ED disposition (discharge, admission to ICU, admission to non-ICU, other). Safety outcomes assessed during DKA treatment included incidence of hypoglycemia (glucose <70 and <54 mg/dL, which have been defined as hypoglycemia and clinically important hypoglycemia warranting reporting in clinical trials, respectively), and incidence of hypokalemia (K < 3.3 mmol/L) and severe hypokalemia (K < 3.0 mmol/L).^9^

The time from ED arrival to administration of long-acting subcutaneous insulin (eg, glargine) marked the duration of “DKA treatment” for these safety outcomes, as this transition to long-acting insulin coincides with DKA resolution. The insulin infusion stop time was used as the end time if no long-acting insulin was given, and 24 hours after ED arrival was used as the end time if insulin infusion stop time was missing, which is consistent with the definition of DKA treatment duration used in previous retrospective studies.^6^ ^,^ ^8^ Chart review was conducted for euglycemic patients to identify the most likely etiology for their euglycemia based on ED documentation (insulin administration prior to arrival, SGLT2 inhibitor use, etc). This review was done by a single author who was not blinded to the study hypothesis and did not receive specific training, and we did not assess interobserver reliability.^10^

The management of DKA in our ED and ED-ICU has been standardized using the two-bag method. This consists of a fixed-rate IV insulin infusion, constant fluid and electrolyte delivery but titratable dextrose delivery, and frequent lab draws with a nurse-driven fluid titration and electrolyte replacement protocol. With resolution of DKA, defined as pH >7.30, serum bicarbonate >15 mmol/L, glucose <200 mg/dL, anion gap <12, and ability to tolerate by mouth, patients are given subcutaneous insulin with two hours of IV insulin infusion overlap prior to discontinuing the insulin infusion.

### Statistical Analysis

For comparisons between the hyperglycemic DKA and euglycemic DKA groups we used independent-groups *t*-tests for continuous variables and chi-squared tests for binary variables. We used an alpha level of 0.05 for all analyses; all hypothesis tests were two-sided, and *P*-values for all test statistics were calculated based on cluster-robust standard errors adjusted for multiple visits clustered within patients. We conducted analyses with the Stata software package (StataCorp LLC, College Station, TX).^11^

## RESULTS

We identified 629 adult ED patients with DKA managed from 2015–2022, 44 of whom were euglycemic (initial glucose ≤250 mg/dL) on presentation and 585 hyperglycemic. Patient demographics and presenting metabolic derangements are summarized in Table 1. Mean age was 31.1 vs 39.8 (*P* < 0.001), and gender was 68.2 vs 60.5% female. At the time of ED presentation, mean blood glucose was 195 vs 561 (*P* < 0.001). Euglycemic patients had milder DKA on presentation with pH of 7.17 vs 7.14, bicarbonate of 11.9 vs 10.4, and anion gap of 21.6 vs 26.3 (*P*s < 0.05). Presenting potassium was significantly lower in euglycemic patients, 4.3 vs 5.3 (*P* < 0.001).

**Table 1.** Patient demographics.

Patient demographics	Hyperglycemic DKA (*n* = 585)^a^	Euglycemic DKA (*n* = 44)^b^	*d* (95% CI)	*P*
Mean age, years (95% CI)	39.8 (36.9, 42.7)	31.1 (27.1, 35.1)	8.7 (4.6, 12.9)	<.001
Female gender, n (%)	354 (60.5)	30 (68.2)	−7.7 (−2.2, 6.6)	0.29
Mean weight, kg (95% CI)	72.0 (68.5, 75.6)	70.2 (63.5, 76.9)	1.8 (−4.5, 8.2)	0.57
Mean presenting laboratory values (95% CI)				
pH	7.14 (7.13, 7.15)	7.17 (7.14, 7.19)	−.03 (−.06, −.004)	0.03
Bicarbonate (mmol/L)	10.4 (9.9, 10.9)	11.9 (10.7, 13.0)	−1.4 (−2.6, −0.2)	0.02
Anion gap	26.3 (25.6, 27.0)	21.6 (19.8, 23.4)	4.7 (2.9, 6.5)	<.001
Glucose (mg/dL)	561 (531, 592)	195 (182, 208)	367 (334, 399)	<.001
Potassium (mmol/L)	5.3 (5.1, 5.4)	4.3 (4.1, 4.6)	1.0 (0.7, 1.2)	<.001

^a^ *n* = 585 visits from 370 patients.

^b^ *n* = 44 visits from 38 patients.

*DKA*, diabetic ketoacidosis; *d*, absolute difference; *C*I, confidence interval; *mmol/L*, millimoles per liter; *mg/dL*, milligrams per deciliter.

Resource utilization outcomes are presented in Table 2. The mean time on IV insulin infusion was significantly shorter at 13.5 vs 19.5 hours (*P* = 0.003), whereas the mean time until normalization of serum bicarbonate >18 mmol/L (12.3 vs 12.1 hours) and time to first long-acting subcutaneous insulin (16.7 vs 16.0 hours) were not significantly different. Total hospital LOS was shorter for euglycemic patients at 2.2 vs 3.9 days (*P* = 0.03), whereas mean ED-ICU LOS was similar (16.4 vs 16.6 hours). Admission rates to ICU were 0% vs 5.1%, and ED discharge rates were 57% vs 33% for euglycemic and hyperglycemic patients, respectively.

**Table 2.** Resource utilization.

Resource utilization	Hyperglycemic DKA (*n* = 585)^a^	Euglycemic DKA (*n* = 44)^b^	*d* (95% CI)	*P*
Mean time on insulin infusion, hours (95% CI)	19.4 (17.6, 21.3)	13.5 (10.1, 16.9)	5.9 (2.1, 5.8)	0.003
Mean hours to first bicarbonate >18 mmol/L (95% CI)	11.9 (11.2, 12.5)	12.3 (10.6, 14.1)	−0.5 (−2.2, 1.2)	0.59
Mean time to first long-acting subcutaneous insulin administration, hours (95% CI)	16.7 (16.0, 17.5)	16.0 (14.4, 17.6)	0.7 (−1.1, 2.5)	0.43
Mean total length of stay, days (95% CI)	3.9 (3.3, 4.4)	2.2 (0.8, 3.6)	1.7 (0.2, 3.1)	0.03
Mean ED-ICU length of stay, hours (95% CI)	16.6 (15.8, 17.4)	16.4 (14.8, 18.1)	0.2 (−1.7, 2.0)	0.85
Emergency department disposition, n (%)				
Admit to ICU	30 (5.1)	0		
Admit to non-ICU	356 (60.9)	19 (43)		
Discharge	193 (33)	25 (57)		
Deceased	1 (0.17)	0		
Other (left against medical advice, send to operating room, transfer to another facility, send to psychiatric emergency department)	5 (0.85)	0		

^a^ *n* = 585 visits from 370 patients.

^b^ *n* = 44 visits from 38 patients.

*DKA*, diabetic ketoacidosis; *d*, absolute difference; *CI*, confidence interval; *mmol/L*, millimoles per liter; *ED*, emergency department; *ICU*, intensive care unit.

Key safety outcomes are shown in Table 3. There was a significantly higher rate of hypoglycemia (<70 mg/dL), 18.2 vs 4.6% (*P* = 0.02), and trends toward more clinically important hypoglycemia (<54 mg/dL), 4.5 vs 1.9% (*P* = 0.40),in the euglycemic cohort. The rates of hypokalemia (<3.3 mmol/L) and severe hypokalemia (<3.0 mmol/L) were not significantly different at 27.3 vs 19.1% (*P* = 0.23), and 6.8 vs 6.3% (*P* = 0.94). Hospital mortality was low in both groups at 0 vs 0.9% in euglycemic and hyperglycemic cohorts.

**Table 3.** Safety outcomes.

	Hyperglycemic DKA (*n* = 585)^a^	Euglycemic DKA (*n* = 44)^b^	*d* (95% CI)	*p*
Hypoglycemia incidence, n (%)				
Glucose <70 mg/dL	28 (4.8)	8 (18.2)	−13.4 (−24.7, −2.1)	0.02
Glucose <54 mg/dL	11 (1.9)	2 (4.5)	−2.7 (−8.8, 3.6)	0.40
Hypokalemia incidence, n (%)				
Potassium <3.3 mmol/L	112 (19.1)	12 (27.3)	−8.1 (−21.4, 5.2)	0.23
Potassium <3.0 mmol/L	38 (6.5)	3 (6.8)	−0.3 (−8.2, 7.5)	0.94
Potassium <2.7 mmol/L	14 (2.4)	1 (2.3)	0.1 (−4.5, 4.7)	0.96
Admission to non-ICU with transfer to ICU within 24 hours, n (%)	1 (0.2)	0	*na*	*na*
Discharge from ED with return and readmission within 72 hours, n (%)	10 (1.7)	0	1.7 (0.5, 2.9)	0.006
Hospital mortality, n (%)	5 (0.9)	0	*na*	*na*

^a^ *n* = 585 visits from 370 patients.

^b^ *n* = 44 visits from 38 patients.

*DKA,* diabetic ketoacidosis; *d*, absolute difference; *CI*, confidence interval; *mg/dL*; milligrams per deciliter; *mmol/L*, millimoles per liter; *ED*, emergency department; *ICU*, intensive care unit.

Of the 44 patients who were euglycemic on presentation, etiologies of euglycemia are provided in Table 4. The majority of etiologies, 86%, were related to insulin use and poor oral intake prior to arrival, with only 14% related to SGLT2 inhibitor use.

**Table 4.** Etiologies of euglycemia.

Etiologies of euglycemia	**n**	**%**
SGLT2 use	6	14
Insulin prior to arrival	25	57
Insulin pump	5	11
Poor oral intake	8	18

*SGLT2*, sodium-glucose co-transporter 2.

## DISCUSSION

In this study, we present clinical data, resource utilization, and safety outcomes in 629 adult ED patients with DKA, 44 of whom were euglycemic on arrival. Euglycemic patients had overall milder DKA on presentation, with higher pH and bicarbonate and lower anion gaps. We observed a shorter mean time of IV insulin infusion for euglycemic patients (13.5 vs 19.5 hours); however, there was no difference in mean time until bicarbonate >18 mmol/L or mean time to first long-acting subcutaneous insulin between cohorts. This suggests that patients with hyperglycemic DKA may have been continued on insulin infusions based on continued hyperglycemia as opposed to resolution of acidosis.

We also observed shorter total hospital LOS (2.2 vs 3.9 days) among patients with euglycemic DKA, although without significant differences in ED-ICU LOS. This suggests that the primary driver of increased LOS lies beyond the initial resuscitation and resolution of DKA, which was done primarily in the ED-ICU. This is also reflected by the reduced rates of ICU admission (0% vs 5.1%) and increased rates of ED discharge (57% vs 33%) for euglycemic patients. We hypothesize that this may reflect an association with more severe underlying triggers or stressors precipitating hyperglycemic DKA (ie, infection, ischemia, shock) that require additional time and level of care to address as an inpatient after the initial DKA resuscitation.

Importantly, we observed increased rates of hypoglycemia (<70 mg/dL), 18.2 vs 4.6%, and trends toward more clinically important hypoglycemia (<54 mg/dL), 4.5 vs 1.9%, in euglycemic patients. Hypoglycemia is well known to have neurological manifestations causing coma and seizures in the acute setting, in addition to being associated with higher rates of strokes and cognitive decline in the long term with repeated episodes.^12^ Hypoglycemia also acutely increases the risk for life-threatening bradyarrhythmias and tachyarrhythmias due to depolarization and repolarization abnormalities and increased ectopy stemming from alterations in sympathoadrenal activity.^12^ ^,^ ^13^ Iatrogenic hypoglycemia is a crucial adverse event to avoid during management of DKA, and our data suggests that standard DKA treatment protocols may require adjustment and closer glucose monitoring for patients presenting with euglycemic DKA. Future studies can investigate protocol adjustments such as higher concentrations of dextrose while on insulin infusion for patients with euglycemic DKA.

Euglycemic DKA has gained significant recognition after the introduction of SGLT2 inhibitors and their relationship with this condition; however, there is minimal data on the epidemiology of the various causes of euglycemic DKA.^3^ We observed only 14% of our euglycemic DKA cohort was taking SGLT2 inhibitors, as opposed to the remaining 86% whose euglycemia was attributable to a combination of exogenous insulin use and starvation state prior to arrival. This includes insulin self-administered by patients, insulin given by emergency medical services or other outside medical professionals, and insulin pump usage. In most cases the insulin was given subcutaneously immediately prior to leaving for the hospital or en route to the hospital upon patient or caregiver recognition of hyperglycemia. Although SGLT2 inhibitor use is an important cause of euglycemic DKA and becoming more widespread, our data notably shows a low prevalence of SGLT2 inhibitor use among our euglycemic DKA patients. Clinicians should maintain a high suspicion for euglycemic DKA in patients taking these medications, but they should not discount the possibility of euglycemic DKA in patients who are not taking these medications.

There are minimal prior studies describing the care of patients with euglycemic DKA beyond case reports and series related to SGLT2 inhibitor use.^14^ ^–^ ^19^ We present the largest cohort to date of ED patients presenting with euglycemic DKA from a variety of causes, which contributes to increased generalizability. This is also the first direct cohort comparison of patients with euglycemic vs hyperglycemic DKA. This data can help guide emergency clinicians when attempting to diagnose and treat patients with euglycemic DKA and continue to advance the field in caring for this important and growing patient population.

## LIMITATIONS

This study was conducted in a unique ED-ICU setting at a single academic medical center in the United States, which may make reproducibility to other settings uncertain. An automated EHR search was used to collect retrospective data for this study and, thus, data points may be prone to human entry error (eg, time of insulin infusion start and stop times). The DKA order set at our hospital has and continues to undergo iterative minor changes, making it possible that safety outcomes measured in this study could have differed over time based on clinical experience and fine tuning of the order set. The sample size of 44 euglycemic patients in this study—while the largest reported cohort of euglycemic DKA ED patients—is relatively small compared to the hyperglycemic cohort (585 patients), which may have increased the possibility of chance contributing to the results and thus the comparisons drawn. Etiologies of euglycemia were discerned from manual chart review by a single author. This left potential for subjectivity in determining the most likely factor contributing to euglycemia for a given patient.

## CONCLUSION

We present key clinical and demographic data as well as safety outcomes in 44 adult ED patients with euglycemic DKA and compare it to those with hyperglycemic DKA managed during the same period. Euglycemic DKA patients had milder DKA on presentation based on pH, bicarbonate, and anion gap, were on insulin infusions for shorter amounts of time, had shorter total hospital LOS, and notably had significantly higher rates of hypoglycemia during treatment. The majority of cases of euglycemic DKA were related to insulin use prior to arrival, with only 14% related to SGLT2 inhibitor use. Euglycemic DKA is an important clinical entity that can be difficult to diagnose and requires thoughtful management to avoid adverse events. Future work can investigate treatment strategies for euglycemic DKA to help minimize the rate of adverse events, especially iatrogenic hypoglycemia.
